# Stronger responses in the visual cortex of sighted compared to blind individuals during auditory space representation

**DOI:** 10.1038/s41598-018-37821-y

**Published:** 2019-02-13

**Authors:** Claudio Campus, Giulio Sandini, Maria Bianca Amadeo, Monica Gori

**Affiliations:** 1 U-VIP Unit for Visually Impaired People, Fondazione Istituto Italiano di Tecnologia, Via Morego 30, 16132 Genova, Italy; 2 RBCS Robotics, Brain and Cognitive Sciences department, Fondazione Istituto Italiano di Tecnologia, Via Morego 30, 16132 Genova, Italy; 3 Università degli studi di Genova, Department of Informatics, Bioengineering, Robotics and Systems Engineering, Via all’Opera Pia 13, 16145 Genova, Italy

## Abstract

It has been previously shown that the interaction between vision and audition involves early sensory cortices. However, the functional role of these interactions and their modulation due to sensory impairment is not yet understood. To shed light on the impact of vision on auditory spatial processing, we recorded ERPs and collected psychophysical responses during space and time bisection tasks in sighted and blind participants. They listened to three consecutive sounds and judged whether the second sound was either spatially or temporally further from the first or the third sound. We demonstrate that spatial metric representation of sounds elicits an early response of the visual cortex (P70) which is different between sighted and visually deprived individuals. Indeed, only in sighted and not in blind people P70 is strongly selective for the spatial position of sounds, mimicking many aspects of the visual-evoked C1. These results suggest that early auditory processing associated with the construction of spatial maps is mediated by visual experience. The lack of vision might impair the projection of multi-sensory maps on the retinotopic maps used by the visual cortex.

## Introduction

Space representation is one of the hardest problems the brain has to solve, given the multiple and diverse information it is constantly receiving by our senses (for review see^1–3^). Vision is the sensory modality which is mostly based on a retinotopic representation of space. Nevertheless, the role of visual information on the development of spatial abilities of other sensory modalities, such as audition and touch, is still a matter of debate. Studying blindness offers valuable insight into this topic.

Several studies have highlighted enhanced auditory processing in blind individuals, suggesting that they partially compensate for their visual impairment with greater sensitivity of the other senses. The visual cortex is highly plastic, particularly in young animals, and retains a good deal of plasticity even in adulthood (see^4^). This plasticity allows the visual cortex of congenitally blind individuals to become to a certain extent colonized by the auditory and somatosensory systems (e.g.^5,6^). As a result, a strong and reliable response to sound presented alone has been observed in the primary visual cortex of blind individuals with fMRI^7–10^ and Event Related Potentials (ERPs^11,12^). Moreover, recent findings show that the retinotopic organization of the human visual cortex is to a certain extent preserved in blind individuals^13^ and in sight-recovery ones^14^. There is also psychophysical evidence that congenital blind subjects have enhanced tactile discrimination^15^, auditory pitch discrimination^16^, sound localization^17,18^, and they are able to properly form spatial topographical maps (e.g.^19,20^).

On the other hand, it is well known that auditory spatial maps can be modified by vision, suggesting that the visual system may be important for auditory spatial perception. Biases in auditory localization have been shown in owls reared with distorting prisms^21^, and total visual deprivation of young ferrets has been shown to affect the development of auditory spatial maps^22^. Comparable (but transitory) effects have also been observed in humans^23,24^. In particular, recent psychophysical studies suggest that early visual deprivation negatively impacts on several auditory spatial localization skills. An example is the audio spatial bisection task, for which blind humans are strongly impaired^25,26^. Contrarily to other tasks (e.g.^16–18,27^) which do not require estimation and comparison of spatial positions, the bisection task taxes a metric representation of the auditory space. The subject needs to judge the relative position of a sound source in a sequence of three spatially separated sounds. We have previously used this task to study the typical development of auditory space representation^28^, showing that children as young as six years of age perform well, with thresholds only slightly higher than adults.

Recently, we investigated the neural cortical correlates associated with the spatial bisection task in sighted subjects. Sighted subjects, who succeed at the space bisection task, are characterized by an early response of visual cortex (P70) during space but not during time bisection task^29^. Specifically, during space bisection task, a P70 component has been elicited by the second sound in a [50–90 ms] time window, which represents a key time window in the earliest stages of multisensory integration. Interestingly, a similar activation was absent during an auditory time bisection task, which requires the evaluation of time intervals between three consecutive sounds. In sighted people, the early occipital response appears strong and contralateral to the sound position in space, thus resembling the C1 ERP component - which is typically observed during visual processing^30^. Beyond this study, other research has previously shown a response of the primary visual cortex to purely auditory stimuli, for instance in mice^31^, cats^32^, primates^33–35^, and even in humans using fMRI^36–39^, TMS^40^ and ECoG^41^ and EEG techniques^42^. In particular, a recent ERP study reported that auditory stimuli can elicit a later contralateral occipital ERP response (ACOP) in sighted individuals^43–46^. ACOP originates from the ventrolateral extrastriate visual cortex (BA 19) and occurs in a late [250–400 ms] post-stimulus time window. Furthermore, audio-visual neuronal interactions have been demonstrated in the early visual cortex. For example, different studies have reported an amplification/reduction of the visual response based on the existence of congruency between visual and auditory signals^36,42,47–50^, and the encoding by the early visual cortex of the abstract information of acoustic object shapes^31–35,37,39^. Taken together, these findings point to an important role of the visual cortex in audio-visual integration^40,51–56^.

While neural correlates for audio enhancement in blind individuals have been extensively explored, the neural correlates behind the audio impairment during the spatial bisection task are still unknown. If the visual cortex has an important role for the audio spatial bisection task in sighted people^29^, we may expect that the deficit observed in blind individuals for this task^25^ might be related to a different processing of audio spatial representations in these cortical regions. Here, we tested this hypothesis by studying the neural correlates associated with audio spatial bisection task in blind individuals.

In this work, ERPs and psychophysical responses were recorded for sighted and blind subjects during an auditory spatial bisection task. Results confirm our hypothesis by showing a stronger visual cortical activation in sighted than in early blind subjects, thus reflecting a key relationship between visual deprivation and human ability to build spatial metrics^25^. Specifically, the lack of vision seems to affect the projection of spatial representations on the retinotopic maps of the visual cortex.

## Results

### Sensor Level Analysis

**Figure 1:**
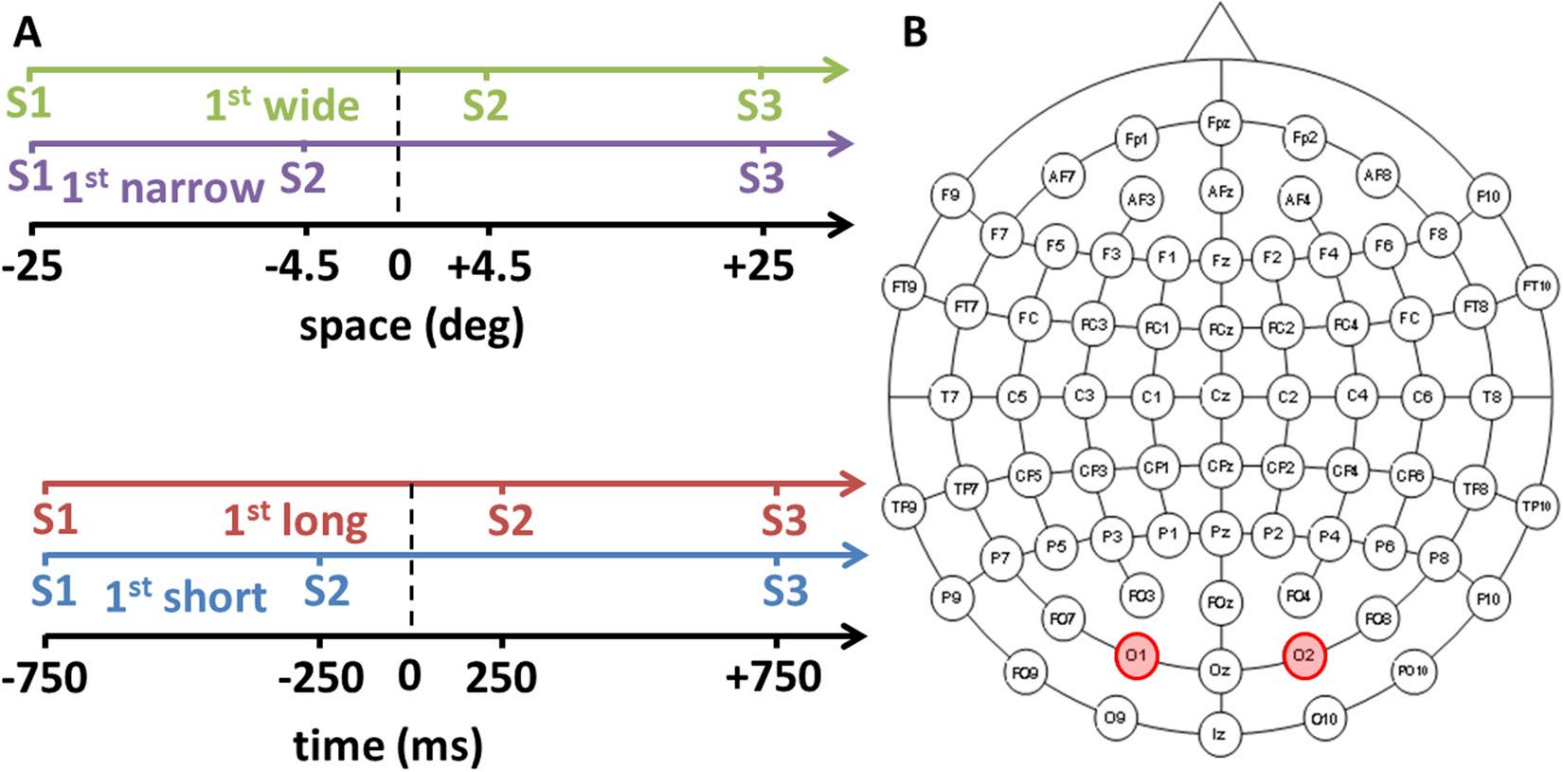
Auditory spatial or temporal bisection. (**A**) Experimental design. Participants listened to three consecutive auditory stimuli (S1, S2, S3) delivered from the lower visual hemifield which lasted 1.5 sec. S1 and S3 were always delivered at ± 25° with respect to the subject midline. S2 could be delivered independently and randomly from ± 4.5° (top) and ± 250 ms (bottom) from the physical spatial and temporal mid points (dashed vertical line). S2 was also presented at 0° and at 0 ms during catch trials to avoid stereotypical responses. Subjects were asked to evaluate whether the position of S2 in space (i.e. spatial bisection block) or time (i.e. temporal bisection block) was farther from S1 or S3. (**B**) Electrode montage used for EEG recording and electrodes considered in the analysis. In red, left (O1) and right (O2) occipital electrodes.

Sixteen blindfolded sighted individuals and sixteen early blind subjects performed two auditory bisection tasks (Fig. 1A) on a sequence of three sounds (S1, S2, S3), where S2 was independently presented at two different spatial positions along a horizontal axis, and with two different temporal lags. Subjects were asked to compare the first distance/interval (i.e. between S1 and S2) with the second distance/interval (i.e. between S2 and S3) and report whether the first distance/interval was temporally longer (i.e. temporal bisection task) or whether it was spatially wider (i.e. spatial bisection task) than the second distance/interval. In the case of the space bisection task, distances reflected the spatial positions of S2: narrow/wide first distances corresponded to S2 delivered from the left (−4.5°)/right (+4.5°) side of the subject respectively. The spatial and temporal position of S2 with respect to the physical bisection position was determined based on thresholds derived from a preliminary study on a different sample: 4^o^ ± 2 (mean ± SEM) for space bisection, and 250 ± 15 ms for temporal bisection.

First of all, we confirmed our previous psychophysical study^25,26^ showing a deficit in blind individuals in performing a spatial bisection task. Compared with sighted (S), early blind participants (B) showed (mean ± SEM) lower probability of correct response (B = 65.6 ± 1.9, S = 88.5 ± 2.2, t_15_ = −11.32, P = 10^−8^) and similar execution times (B = 1.44 ± 0.33, S = 0.94 ± 0.13, t_15_ = 1.38, P = 0.19). The deficit was not present in the temporal bisection task, for which the probability of correct response (B = 82.8 ± 3.8, S = 83.7 ± 2.7 t_15_ = −0.29, P = 0.77) and execution times (B = 0.97 ± 0.10, S = 0.95 ± 0.12, t_15_ = 0.02, P = 0.99) were not significantly different between the two groups.

**Figure 2:**
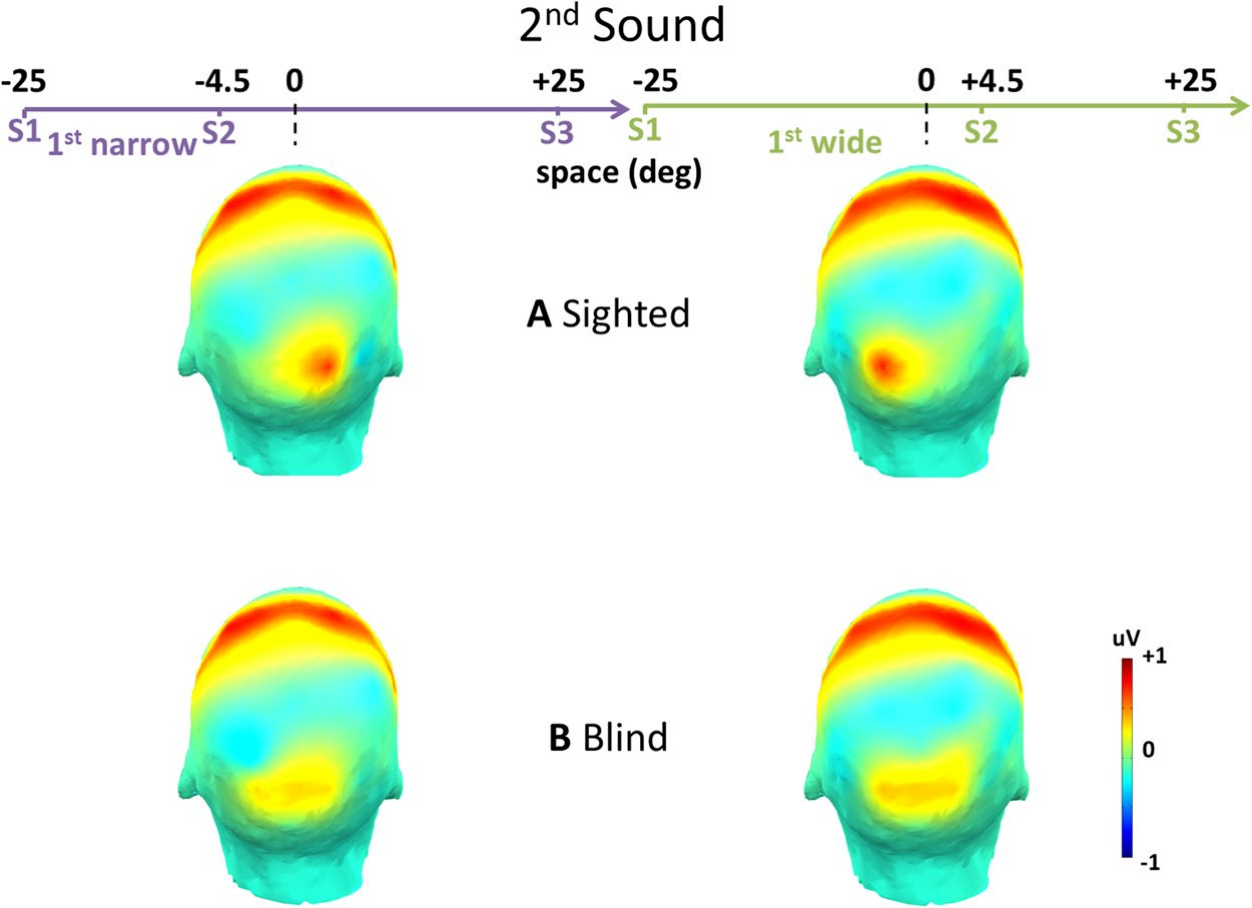
ERP topography, i.e. scalp map of the mean amplitude in the [50–90 ms] time window averaged across subjects for sighed (**A**) and blind (**B**) groups (no masking is applied): the effect of S2 during space bisection. Left and right panels of the figure report the conditions in which the first distance was narrow (i.e. S2 delivered from the left, at −4.5°) or wide (i.e. S2 from the right, at +4.5°) respectively. Two strong positivities appeared. One involves central areas, and one involves parieto-occipital areas. This latter positivity showed a specific contralaterality during space bisection task only in sighted subjects (**A**). In blind participants (**B**), the parieto-occipital response was strongly attenuate and not contralateral to S2 spatial position.

More importantly, the present paper focuses on event related potentials (ERPs) elicited by S1 taken as a control, and by S2, which is necessary for the construction of a spatial or temporal metric. ERP amplitude was obtained averaging the response within the C1 time window. Time window (50–90 ms) and topography (O1, O2) were selected based on the grand average merging all conditions^29,57^. A first omnibus ANOVA on the mean ERP amplitude in the C1 [50−90 ms] time window showed a strong interaction between Sound (S1, S2), Domain involved by the bisection task (Space, Time), Hemisphere (Left, Right), First distance/interval extension (Narrow/Short, Wide/Long) and Group (Blind, Sighted; F_1,30_ = 33.11, P = 0.000003, generalized eta squared (ges^58^) = 0.51). We subsequently performed hypothesis-driven follow-up ANOVAs and post hoc comparisons. First, we hypothesized that S2 could specifically modulate the interaction between other factors. Therefore, we performed two separate ANOVAs (one for each sound), with Domain, Hemisphere and First distance/interval extension as within subject factors, and Group as between subject factor. As expected, we found a significant interaction between Domain, Hemisphere, First distance/interval extension and Group for S2 (F_1,30_ = 35.04, P = 0.000002, ges = 0.43). On the contrary, for S1 we found only an expected main effect of the Hemisphere, given that S1 way always played from the left (−25°; F_1,30_ = 4.75, P = 0.03, ges = 0.19). Thus, we focused analyses on S2, separately evaluating the two domains (Space, Time). Therefore we performed two separate ANOVAs (one for Space, the other for Time), with Hemisphere and First distance/interval extension as within subject factors, and Group as between subject factor. For the space domain, we found a significant interaction between Hemisphere, First distance/interval extension and Group (F_1,30_ = 37.21, P = 0.000001, ges = 0.43), while for the time domain we did not find any significant interaction (F_1,30_ = 0.18, P = 0.67, ges = 0.001). Post-hoc analyses involved only S2 in the space domain, revealing a stronger lateralized P70 in sighted compared to blind subjects. Figure 2 illustrates the scalp maps elicited by S2 delivered from −4.5°(left panel) and + 4.5° (right panel) in the P70 (50–90 ms) time window during the spatial bisection task, for sighted (Fig. 2A) and blind participants (Fig. 2B). A positivity is evident in occipital areas for both groups. However, we can observe that in visually deprived individuals the occipital positivity resulted attenuated in sites contralateral with respect to the S2 position in space and increased in ipsilateral ones. Specifically, when the first distance was narrow (S2 delivered from left, −4.5°), the contralateral electrode O2 showed a strongly higher response in sighted individuals (t_30_ = 6.21, P = 0.00000008), while the ipsilateral electrode O1 showed a moderately higher response in blind individuals (t_30_ = 3.82, P = 0.0006). Symmetrically, when the first distance was wide (S2 from right, +4.5°) the contralateral electrode O1 showed a strongly higher response in sighted subjects (t_30_ = 6.96, P = 0.00000001), while the ipsilateral electrode O2 showed a moderately higher response in blind subjects (t_30_ = 3.70, P = 0.0009).

**Figure 3:**
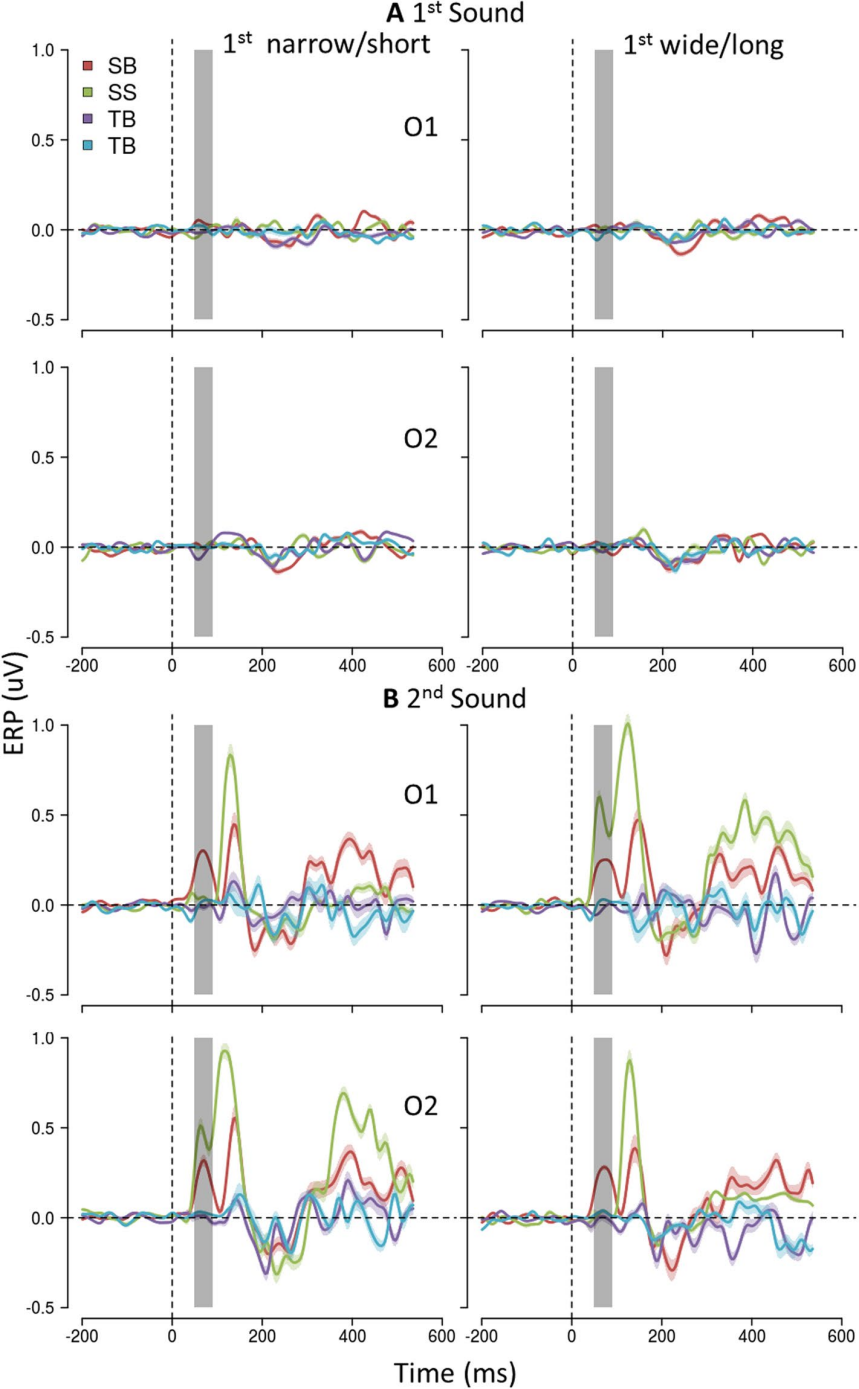
Modulation of the occipital ERP response due to S2 physical position during spatial and temporal bisection task. ERPs (mean ± SEM) in O1 (first row) and in O2 (second row) after S1 (**A**) and S2 (**B**) are reported separately for each group (sighted S/blind B) and task (time bisection: TS, TB /space bisection: SS, SB). On the left, trials in which S2 was delivered from the left hemispace (space bisection), or with shorter temporal separation from S1 (time bisection), giving rise to a narrow first distance/short first interval. On the right, trials in which S2 was delivered from the right hemispace (space bisection), or with longer temporal separation from S1 (time bisection), given rise to a wide first distance/long first interval. Physical position of S1 (**A**) does not modulate occipital response in O1 and O2 during neither the space nor the time bisection task. Physical position of S2 (**B**) modulates the occipital response of sighted individuals in O1 and O2 during the space bisection task. t = 0 is sound onset. Shaded areas delimit P70 (50–90 ms) time window.

As expected^29^ (see Fig. 3 and Supplementary Fig. 1), only the spatial but not the temporal bisection task elicited the specific early occipital response. Moreover, any specific early occipital response to S1 appeared neither for sighted nor for blind subjects, independently of the task domain. Interestingly, for both sighted and blind subjects we found a later response (P140, see Supplementary Fig. 2), selective again for S2 during the space bisection task but without any lateralization effects. P140 was more pronounced in sighted subjects, probably reflecting a stronger activation of an extended dorsal stream. The time window considered in the analyses was the first one presenting a task-related modulation (see Fig. 3), while a later activation seems to occur resembling the ACOP component (see Supplementary Fig. 3), reported in sighted individuals within the [250–400 ms] time window by previous studies^43–46^. Like the early response, ACOP resulted more pronounced and lateralized in sighted individuals, while lower and not lateralized in blind participants.

**Figure 4:**
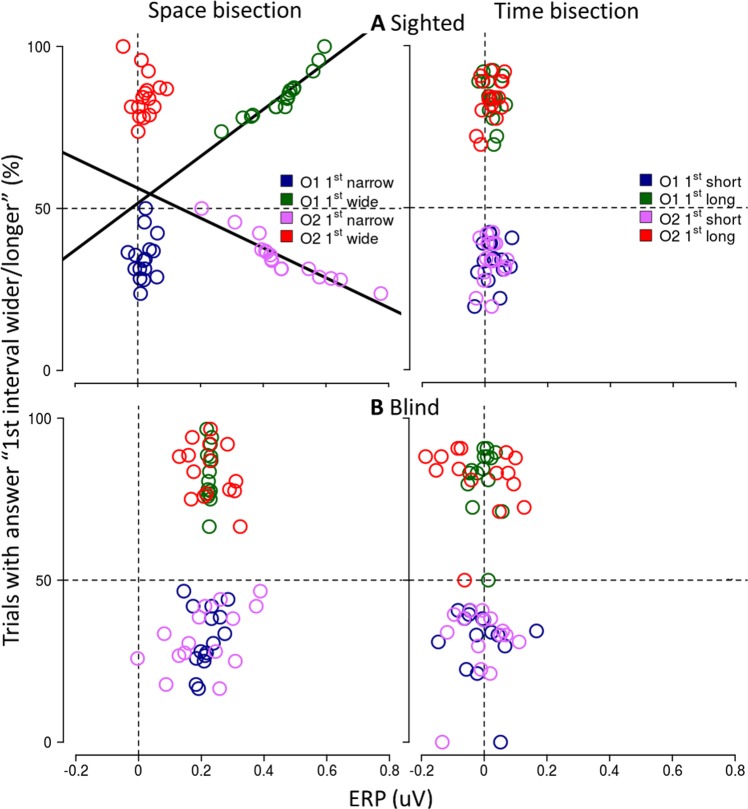
Correlation between the perceived localization of S2 and mean ERP amplitude in the selected time window after S2, evaluated for sighted (**A**) and blind (**B**) group separately. We separately consider the conditions in which the first distance/interval was physically narrow/short and wide/long (respectively, narrow/wide for space bisection, see left panel; short/long for time bisection, see right panel). For each condition and each subject, individual mean ERP amplitude in O1 (blue and green) and O2 (red and pink) is plotted against the percentage of trial in which the subject perceived the first distance/interval as wider/longer, i.e. wider in the spatial bisection task and longer in the temporal bisection task. In space bisection, perceiving narrower and wider first distance corresponded to perceiving S2 delivered from left and right side respectively. Thick black regression lines represent significant correlations. Sighted subjects (**A**) show a specific correlation between perceived localization of S2 during the space bisection task and ERP response in contralateral occipital electrodes.

To verify that [50–90 ms] evoked response is associated with the perceived position of S2 rather than with its physical location, for each physical extension of the first distance/interval (narrow/short, wide/long) we correlated individual ERP responses recorded in O1 and O2 with the individual percentage of trials in which the first distance/interval was perceived as wider/longer (see Fig. 4 and Supplementary Table 1). As shown in Fig. 4A, for sighted subjects the ERP amplitude in O1 and O2 was significantly associated with subject performance in the space but not the time bisection task. Specifically, the percentage of trials in which participants reported the first distance as narrow (i.e. S2 perceived as delivered from the left) and wide (i.e. S2 perceived as delivered from the right) correlates with the ERP amplitude in the occipital contralateral electrodes, O1 and O2 respectively (for O1 and wide first distance R = 0.90 P = 0.000002, for O2 and narrow first distance R = −0.89 P = 0.000004). Instead, blind subjects did not show any similar correlation, during neither the spatial nor the temporal bisection task.

According to our data, the effect does not originate from eye-movements towards the apparent sound position. Indeed, the average ocular deflection recorded by electrooculography (EOG) is equal and not different from zero when grouping according to the position of S1 or S2, for both physical (as for the lowest P value, sighted: t_15_ = 0.62, P = 0.54, blind: t_15_ = 0.82, P = 42) and perceived distance/interval (as for the lowest P value, sighted: t_15_ = 0.72, P = 0.48, blind: t_15_ = 1.11, P = 0.29). For the sake of transparency, in Supplementary Figs 4 and 5 we exhibit ERPs from raw non-cleaned data, confirming similar electrophysiological responses, even though less evident due to the worse SNR.

### Source Level Analysis

**Figure 5:**
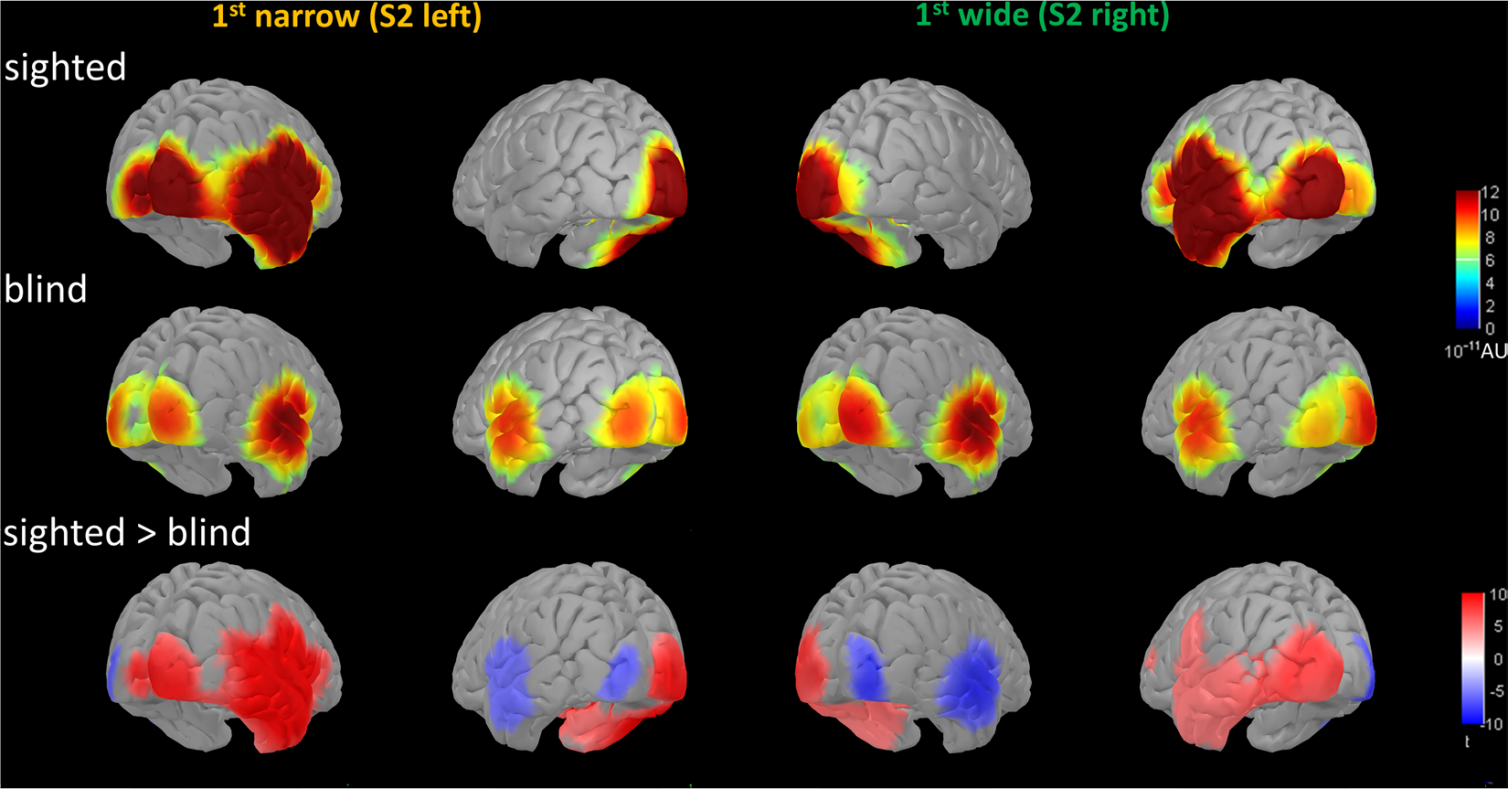
An early activation of the visual cortex contralateral to sound spatial position is elicited by S2 during space bisection in sighted subjects, while it is more attenuated and ipsilateral in blind individuals. Average source activity within the P70 time window (50–90 ms) is compared between sighted and blind subjects. Left and right panels of the figure respectively report the conditions in which the first distance was narrow (i.e. S2 from left) or wide (i.e. S2 from right). The first two lines represent average normalized source activation for sighted (first row) and blind subjects (second row), in arbitrary (normalized) units (AU). No masks have been applied. Last line reports the results of paired two tailed t-tests; the scale is in terms of t-statistic. Significant values of t statistic are displayed: reddish and bluish colours indicate stronger activations in sighted and blind subjects respectively, while the intensity indicates the magnitude of t (i.e. the streght/significance of the difference). Only t values corresponding to p < 0.0001 after FDR correction are displayed.

To test that P70 component was actually involving generators in visual cortex, we compared the two groups at source level (Fig. 5). For each subject and condition, we computed the average source activation within the [50–90 ms] time window and then we calculated the norm of the vectorial sum of the three orientations at each vertex. We used paired t-test to compare these values between groups and we applied FDR correction for multiple comparisons^59^. To verify that the activation after S2 was specific for the spatial bisection task, we compared the sighted with the blind group considering the tasks (space and time bisection), and the sounds (S1 and S2) separately. Only after S2 in the spatial bisection task did we observe significant differences, which are shown in Fig. 5. Left and right parts of the figure report the conditions in which the first distance was narrow (S2 from the left) or wide (S2 from the right) respectively. The first two rows display average normalized source activation of sighted subjects (first row) and blind subjects (second row). The last row shows the result of the t-test comparing groups, displaying signed values of t statistic: reddish and bluish colors mean stronger activations in sighted and blind subjects respectively, while the intensity of the color indicates the significance (strength) of the difference. To minimize the risk of false positives, exclusively t-values corresponding to P < 0.0001 post FDR correction are considered significant and represented.

Thus, comparing groups in the P70 time window during spatial bisection (Fig. 5), sighted subjects showed a stronger occipital and temporal activation contralateral to the spatial position of the sound, while blind subjects exhibited a reduced activation in contralateral cortical areas and an increased activation in ipsilateral cortical areas. These differences between groups were absent after S1 in spatial bisection task, when both groups showed only a similar activation of temporal cortex contralateral to the sound. Similarly, no differences emerged between the two groups considering either S1 or S2 in the temporal bisection task.

## Discussion

This is the first study reporting that early contralateral occipital activation to sound is strong in sighted individuals and dramatically lower in blind individuals. The involvement of striate cortex during unisensory auditory representation has been widely shown in blind subjects^7–12,60^ but more rarely in sighted individuals (e.g. by using fMRI^36^, TMS^40^ and only recently EEG^29^). The difference in the occipital ERP modulation between blind and sighted subjects for our spatial task opens new issues on the strategies and neural circuits underlying the construction of spatial representations mediated by visual experience.

We have recently shown that during a purely auditory spatial bisection task sighted subjects exhibit a strong early occipital response (P70), selective for the sound position^29^. A similar pattern of early contralateral occipital response is generally observed for visual stimuli. The P70 ERP component observed in sighted individuals, resembles the well-known C1 ERP observed during visual tasks^30,61,62^, and it is specifically elicited by the construction of metrics in the space domain^29^. We speculated that this ERP response might be the auditory counterpart of visual C1, reflecting the construction of retinotopic maps in sighted individuals. Here, we report that the same activation is dramatically reduced in contralateral occipital areas of blind individuals, suggesting that the lack of visual experience affects the P70 component.

Moreover, while in sighted individuals we observed a strong correlation between sound localization performance and the mean amplitude of P70 ERP response in contralateral electrodes, the same correlation was not evident in blind individuals. This indicates that in sighted individuals the spatial selectivity of the early occipital response is closely related with the perceived, and not only with the physical, spatial position of sounds. The fact that in blind individuals this laterality is absent means that early visual experience mediates the development of this contralateral early occipital response. In the present paper, the first and the last sound of the bisection task were always played from the left and right side respectively, with the second sound varying its spatial position from trial to trial. The decision of delivering the first and the last sound from fixed position derives from previous studies showing a gross *deficit* of blind individuals in audio spatial bisection^25,26^ using exactly the same paradigm. Although future studies could benefit from a counterbalanced design in which these sounds could be swapped in half the trials, we replicated the results in order to investigate the neural correlates behind the impairment. The neural correlate of this deficit might correspond to the attenuated early occipital activation in contralateral sites and the increased early occipital activation in ipsilateral sites observed here in early blind individuals^25^. Thus, our data suggest that the cortical activation generating the C1 component may be crucial for the construction of spatial metrics, regardless of the involved sensory modality. The spatial metric would then depend on visual experience. Interestingly, differently, from the N48 previously observed in parietal areas during multisensory tasks^63^, the response observed here in sighted but not in blind individuals shows generators in the occipital area^29^. It could be that N48 reflects more multisensory integration processes, whereas the response we observed in the visual cortex of sighted individual is specifically related to the construction of metrics in the space domain.

Our results do not reflect a specific deficit at central auditory location or effects due to enhanced peripheral auditory processing in blind individuals^18^: we previously showed^25^ that blind people can locate single sounds acoustically identical to S2 similarly to sighted people, even for angles as small as those used in this study (±4.5°). As well, our effect does not originate simply from different auditory attentional skills to left or right positions of sounds, or from different auditory attentional skills between sighted and blind individuals. In fact, we found a contralateral activation in temporal electrodes and auditory cortices as expected for the processing of auditory stimuli^29,64^ in both groups, but a similar contralaterality in the visual cortex was observed specifically in sighted individuals. Similarly, attention to space can be expected to weakly affect early ERPs, such as the observed occipital response and the N1a^18,65^. Morover, our data do not reflect a mere indirect auditory activation mediated by the acoustic thalamus specific for sighted individuals^66^: during the time bisection task we did not find any strong early occipital responses in both sighted and blind participants. This could theoretically result from out-of-phase summation of components with opposite polarities but we can exclude this simple summation effect because we found similar patterns also when splitting data based on the physical positions of S2. Moreover, behavioral performance indicates that there was no deficit in memory *per se* in the group of blind individuals: there was no difference in performance and execution times between sighted and blind participants for the temporal bisection task.

We think that a possible explanation about why different activations between sighted and blind individuals were evident only for the spatial bisection task is that vision might have a pivotal role in auditory spatial perception (e.g.^25,67,68^). Another possible interpretation involves differences in spatial imaging strategies between sighted and blind individuals. In recent years, a growing body of research has employed the mental scanning paradigm as a tool to investigate the metric properties of mental spatial images in the blind population^69–72^. In one study^72^ authors claimed that only blindfolded sighted individuals are able to create metrically accurate spatial representations of small-scale spatial configurations by listening to a verbal description or by exploring the configuration haptically. On the other hand, early blind participants, but not sighted individuals, can generate accurate spatial mental images though locomotor exploration of a full-scale navigable environment. These results highlight that spatial imagery in blind subjects differs from the spatial imagery of sighted individuals^72^. However, we find spatial imagery an unlikely explanation of our results as the observed P70 involves a very early time window (50–90), which more likely reflects perceptual than imaging processes.

The present study provides strong evidence for cross-sensory calibration theory^73^, suggesting that visual information is necessary for normal development of the auditory representation of space. Blind subjects did not show a low performance in all our auditory tasks but only in the spatial bisection task, designed to tax a sophisticated and well-calibrated spatial auditory map of Euclidean relationships. Along these lines, children younger than 12 years of age showed a visual dominance over audition in spatial localization tasks along the horizontal axis, rather than optimal integration like in adults^28^. This result implies that in the developing child calibration of the auditory system by vision is fundamental for the formation of auditory spatial maps. Our results in blind individuals are also in line with results in animal models showing that the visual system is crucial in calibrating auditory localization: owls reared with distorting prisms show systematic and persistent biases in auditory localization^21^; early visual deprivation in ferrets causes disordered development of superior collicular auditory spatial maps^22^; altered vision modifies the developing auditory map^74–76^, and relatively brief periods of adaptation to spatially conflicting visual and auditory stimuli biases auditory localization in adults^23,24^.

As to previous studies showing a preserved retinotopic organization in blind and sight-recovery individuals^13,14^, our results do not disagree with them. On the one hand, Striem-Amit and colleagues^13^ revealed using functional connectivity MRI indices that a certain level of large-scale retinotopic organization is retained in the visual cortex of the blind, while we are focusing on a very early time window (50–90 ms) measured with EEG. It could be that a certain level of retinotopic organization is preserved but attenuated in case of blindness, or it could also be that it is not evident in our data because it does not involve the earliest pathways of visual processing. Accordingly, Sourav and colleagues^14^ demonstrated using EEG that one basic feature of the retinotopic organization (i.e. upper versus lower visual field organization), is present in people who were born with total bilateral cataracts and subsequently underwent cataract-removal surgeries. In this regard, we hypothesize that other features maybe associated with retinotopic organization, such as the emergence of the P70 during complex auditory spatial representation, may need the visual input to develop. Moreover, an alternative explanation could be that the construction of auditory spatial metrics tested here reflects a peculiar ability for which retinotopic organization of the striate cortex does not seem to be a prerequisite.

To conclude, our data suggest a key role of the visual modality in the development of an early occipital response specific for space perception and for auditory stimuli. Due to its peculiar spatial resolution and flexibility, the visual brain of sighted subjects seems to be fundamental in the construction of spatial metrics of the environment. Lack of vision seems to impact on the development of this processing and underlying neural circuits, bringing to an impairment in understanding Euclidean relationships, such as those involved in solving a spatial bisection task.

## Materials and Methods

### Participants

The sample consisted of 16 blindfolded sighted subjects (11F, aged 42 ± 16Y, mean ± SEM), and 16 early blind subjects (11F, 42 ± 15Y, see Table 1 for clinical details). All participants reported normal hearing and gave written informed consent prior to testing. The study was approved by the ethics committee of the local health service (Comitato etico, ASL 3, Genova) and performed according to the Declaration of Helsinki.

**Table 1:** Clinical details of early blind (B) participants.

Participant	Age at test	Gender	Pathology	Age complete blindness
#B1	38	M	Retinopathy of Prematurity	Birth
#B2	25	F	Retinopathy of Prematurity	Birth
#B3	49	M	Retinopathy of Prematurity	Birth
#B4	20	F	Congenital Glaucoma	Birth
#B5	72	F	Depth damage of vision in both eyes	Birth
#B6	52	F	Atrophy of the eyeball	Birth
#B7	38	F	Retinopathy of Prematurity	Birth
#B8	26	F	Retinitis pigmentosa	Birth
#B9	55	M	Uveitis	Birth
#B10	28	F	Retinopathy of Prematurity	Birth
#B11	22	F	Congenital Glaucoma	Birth
#B12	60	F	Atrophy of the eyeball	Birth
#B13	56	M	Congenital glaucoma	Birth
#B14	38	F	Congenital cataracts and malformation of the lens	Birth
#B15	55	M	Retrolental fibroplasia	Birth
#B16	48	F	Retinitis pigmentosa	Birth

The table shows age at test, gender, pathology, and age since subjects became completely blind.

### Stimuli

Three auditory stimuli (namely S1, S2, S3; 500 Hz, 75 ms duration, 60 dB SPL at the subject position) were played at three different space locations (Fig. 1A) and three different time lags (Fig. 1B). Sounds were played in the lower visual hemifield along with a horizontal axis through a set of 23 free-field speakers placed at a distance of 180 cm and spanning ±25° of visual angle (with 0° representing the central speaker, negative values on the left, and positive values on the right). S1 and S3 were always presented at −25° (S1, left side of the subject) and +25° (S3, right side of the subjects) degrees, with time separation fixed and equal to 1.5 seconds. From trial to trial, S2 could occur from either −4.5° or 4.5° in space (Fig. 1A), and independently at either −250 ms or +250 ms in time, considering zero the middle of the temporal sound sequence (Fig. 1B). These values derives from preliminary space and time bisection sessions performed by five subjects, and they correspond to approximately 75% of correct answers. Inter-trial interval was set to 1250 ± 250 ms. Time between sounds was sufficient to ensure a complete decay of the ERP response.

### Procedure

Participants performed a space bisection task and a time bisection task in two separated and randomized blocks. Crucially, identical stimuli were provided in both blocks. During the space bisection block, subjects evaluated whether the first spatial distance (between S1 and S2) was smaller or larger than the second spatial distance (between S2 and S3), referred as “narrow” and “wide” respectively (see Fig. 1A**)**. During the time bisection block, they evaluated instead whether the first temporal interval (between S1 and S2) was smaller or larger than the second temporal interval (between S2 and S3), referred as “short” and “long” respectively (see Fig. 1B). During the experimental session, subjects were sitting in front of the central speaker (0°), and they were warned to maintain a stable head position while fixating straight ahead. Their position, as well as their head orientation and EOG signal, were continuously monitored during the test by the experimenters.

In order to bypass potential confounding electrophysiological modulations due to subject responses, participants were asked to answer immediately after the third sound (i.e. S3). We measured individual performance (the percentage of response ‘wider/longer’ for the first distance/interval was calculated as follow: we calculated the number of trials in which the subject perceived the first distance/interval as wider/longer and we divided this number by the total number of trials), and execution times (i.e. the time between S3 and button press). The latter could not be treated as reaction times as they could be affected by different factors (e.g. strategy) but they were recorded to ensure engagement in the task. For statistical purposes, accuracies (% correct) were Z-transformed.

### EEG acquisition and preprocessing

EEG (64 channels) and EOG were acquired using Biosemi Active 2 EEG System following the same procedure applied in a previous study^29^. First, we filtered EEG between 0.1 and 100 Hz and we identified by visual inspection and consequently removed trials with horizontal ocular movements. Moreover, to exclude that effects were due to ocular movements we also compared the amplitude of ocular movements (left-right EOG difference) between conditions and we did not find any modulation associated with the psychical or perceived position of S2. Then, we removed transient stereotypical (e.g. eye blinks) and non-stereotypical (e.g. movement or muscle bursts) high-amplitude artefacts applying the Artifact Subspace Reconstruction method (ASR)^77^, which is a plug-in for EEGLAB software^78^. ASR computes principal components which then span a lower dimensional subspace. Subspace components are compared to properties/results of decomposition from the baseline EEG (the algorithm identifies components from reference EEG data). From the component’s activations, the root mean square amplitude is estimated, as well as their mean and standard deviation^77,78^. Given these statistics, a threshold matrix is calculated. The components derived during the processing are then compared to this threshold matrix to determine whether their variance lies below the threshold. The reconstruction of data takes place in the subspace. In this study, to identify corrupted subspaces we selected a 500 ms sliding window and a threshold of 3 standard deviations. The threshold was chosen to minimize the influence of occasional large-amplitude noise/artefacts, such as bursts originated from muscle contraction. Moreover, we removed channels correlated less than 0.85 to an estimate based on the other channels, or with line noise relative to signal more than 4 standard deviations from the channel population mean. Then, we removed segments in which the fraction of contaminated channels was higher than 0.25. We kept other parameters as their default.

Furthermore, we cleaned EEG data also with independent component analysis^78^. Two EEGLAB toolboxes (i.e. SASICA^79^ and IC_MARC^80^) were used to identify artefactual components based on quantitative criteria (all parameters were kept as their default). For component rejection, we strictly followed the criteria reported in the validation papers, which are mostly based on abnormal spectra and/or topographies. The joint application of ASR and ICA allowed to obtain a particularly good signal to noise ratio, being complementarily efficient in removing two different kinds of artifacts. Given its application of sliding windows, ASR was especially efficient in removing transient artifact (e.g. short muscle contractions). Instead, ICA was applied to remove stereotyped repeated artifacts (e.g. cardiac or long-lasting muscle activities). After application of ASR, the runica function of the EEGLab toolbox automatically estimated the rank of the data and, when required, performed a preliminary dimensionality reduction with PCA before extracting independent components. We referenced EEG data to the average of the left and right mastoids (TP7 and TP8).

### Sensor Level Analysis

Our main aim was to test the hypothesis that blind and sighted subjects show different early cortical responses during the space bisection task, in particular after S2 which is the starting point to for the construction of a spatial metric. Indeed, we previously showed^29^ that in sighted people only S2 could produce a contralateral occipital activation which mimics the neural activation observed during visual processing. In the present paper, we compared the cortical response of the two groups, considering S2 and S1. This latter was taken as a control, while S3 was excluded since it could imply more complex processes related to the definition of spatial metric^29^.

Thus, we averaged EEG data time-locked with S1 or S2 onset, considering a period of 200 ms before S1 onset as a baseline. Although we verified that time between sounds was sufficient to ensure a complete decay of the ERP, the selection of a clean baseline before S1 ensured to avoid for all sounds any possible (even small) effect due to late cognitive processes related to previous sounds. For each participant, we presented 60 trials per block and condition, and a minimum of 40 trials for each ERP was required after artifact removals. Each block included 15 catch trials with exactly the same space distances and time intervals; the latter were not taken into account for statistical analyses of performances and ERPs. The total number of trials for each ERP was equal to 855, approximately 55 per participant.

As in our previous study^29^, we focused on electrodes linked to visual processing (i.e. O1, O2 in occipital areas), which could show contralateral activations with respect to the spatial position of sounds. A [50–90 ms] post-stimulus time window was selected by extracting the grand average ERP (i.e. merging all conditions), thus avoiding possible biases^57^ and in line with other studies reporting typical C1 ERP component elicited by visual stimuli^30,81–83^. Importantly, the selected electrodes were those reporting the major response in the [50–90 ms] time window, once again considering the grand average merging all conditions. Mean ERP amplitude was calculated averaging the response within the C1 component time window (i.e. 50–90 ms).

For statistical comparisons, analysis of variance (ANOVA) was run considering as factors Sound (S1, S2), Domain involved by bisection task (Space, Time), Hemisphere (Left, Right), First distance/interval extension (Narrow/Short, Wide/Long), and Group (Blind, Sighted). Hypothesis-driven ANOVAs and paired two-tailed t-tests were conducted as post-hoc comparisons with probabilities treated as significant when lower than 0.05 after Bonferroni correction, applied to each subset of post-hoc comparisons separately.

The association between individual performance and ERP was addressed with linear regression of individual mean ERP amplitude in the selected time window against the percentage of trials in which each subject perceived the first distance/interval as wider/longer.

### Source Level Analysis

To investigate the cortical generators of the ERP components influenced by the experimental factors, a distributed sources analysis was performed with the Brainstorm software^84^, following the same procedure described in our previous study^29^. We used standard 1 mm resolution brain of the Montreal Neurological Institute (non-linear average of 152 subjects, processed with FreeSurfer 5.3 ICBM152^85^), we performed forward modelling using three-layer (head, outer and inner skull) symmetric boundary element model (BEM) generated with OpenMEEG^86^, and we estimated source intensities using sLORETA approach^87^. Since individual MRIs were not available, the Brainstorm output using a constrained approach could be unrealistically precise (in terms of visualization). Therefore, to avoid misleading over-interpretation, dipole orientations were let free to assume whichever (unconstrained) orientation instead of fixed them to the cortex surface. We averaged source activation for each subject of the two groups and condition within the selected time windows. Subsequently, we estimated the norm of the vectorial sum of the three orientations at each vertex. In the end, pairwise comparisons were investigated with paired t-test, correcting results for multiple comparisons of source grid points with FDR method^59^, using p = 0.0001 as a threshold. To verify the specificity of the activation after S2 in the space bisection task, we compared sighted with blind group considering the tasks (space and time bisection) and the sounds (S1 and S2) separately.

## Supplementary information

Supplementary information

## Data Availability

The datasets generated during and/or analyzed during the current study are available from the corresponding author on reasonable request.
