# Supplementary material for: Stronger responses in the visual cortex of sighted compared to blind individuals during auditory space representation
Source: Sci Rep. 2019 Feb 13;9:1935. doi: 10.1038/s41598-018-37821-y (PMC6374481; doi:10.1038/s41598-018-37821-y)
Supplement: Supplementary file 1 — Supplementary information [file 41598_2018_37821_MOESM1_ESM.pdf]

Supplementary information

Stronger responses in the visual cortex of sighted compared to blind individuals during auditory space representation

Claudio Campus[1,2], Giulio Sandini[2], Maria Bianca Amadeo[1,3], Monica Gori[1,2]*.

[1]U-VIP Unit for Visually Impaired People, Fondazione Istituto Italiano di Tecnologia, Via Morego, 30 - 16163 Genova (Italy).

[2]RBCS Robotics, Brain and Cognitive Sciences dept., Fondazione Istituto Italiano di Tecnologia, Via Morego, 30 - 16163 Genova (Italy).

[3]Università degli studi di Genova, Department of Informatics, Bioengineering, Robotics and Systems Engineering, Via all'Opera Pia, 13 - 16145 Genova (Italy).

*Correspondence: Monica.Gori@iit.it, Fondazione Istituto Italiano di Tecnologia, Via Morego, 30 - 16163 Genova (Italy).

Sensor level analysis

**bisection task**	**1st distance/interval**	**channel**	**R**	**p**
space	wide	O1	0.9	0.000002
		O2	-0.13	0.63
	narrow	O1	0.07	0.8
		O2	-0.89	0.000004
time	long	O1	0.1	0.71
		O2	0.13	0.32
	short	O1	-0.04	0.88
		O2	0.05	0.85

Supplementary Table 1. Correlation between individual performance (i.e. individual percentage of trials in which the first distance/interval was perceived as wide/long or narrow/short) and individual ERP responses. In the first column the bisection task (spatial or temporal); in the second column the distance/interval between S1 and S2 (wide/long or narrow/short); in the third column occipital channel (O1 or O2); in the fourth and fifth columns respectively the R and p value corresponding to the correlation between individual performance and ERP response (mean ± SEM) in uV within the 50-90 ms time window after S2.

P70

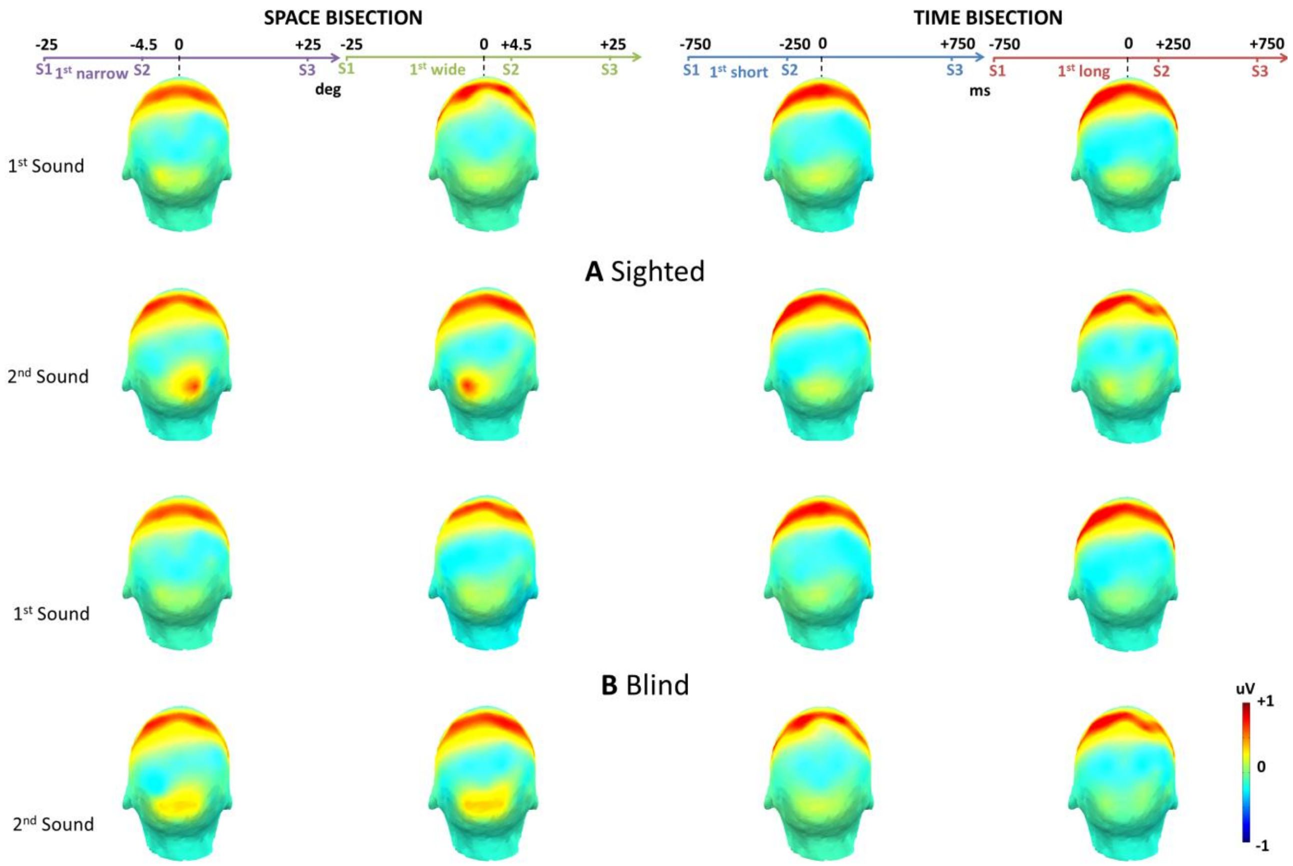

Supplementary Figure 1. ERP scalp map in the P70 time window (50-90 ms) averaged across subjects: the effect of sound and bisection task. The positivity in central areas was not modulated by any experimental factor. In sighted individuals (**A**), the parieto-occipital positivity was contralateral to S2 during the spatial bisection task (bottom left corner of **A**) when a narrow (Left) or a wide (Right) first distance corresponded to S2 delivered from the left or the right hemifield respectively. During the time bisection task (bottom right corner of **A**), neither a short nor a long first interval in the time domain could elicit a similar response. Compared with sighted subjects, blind participants (**B**) showed during the spatial bisection task a lower positivity without any contralaterality (bottom left corner of **B**). The posterior positivity was absent during the time bisection task (right part of **A** and **B**) and after S1 of the spatial bisection task (top left corner of **A** and **B**). Importantly, no other scalp areas were involved by the early positivity.

P140

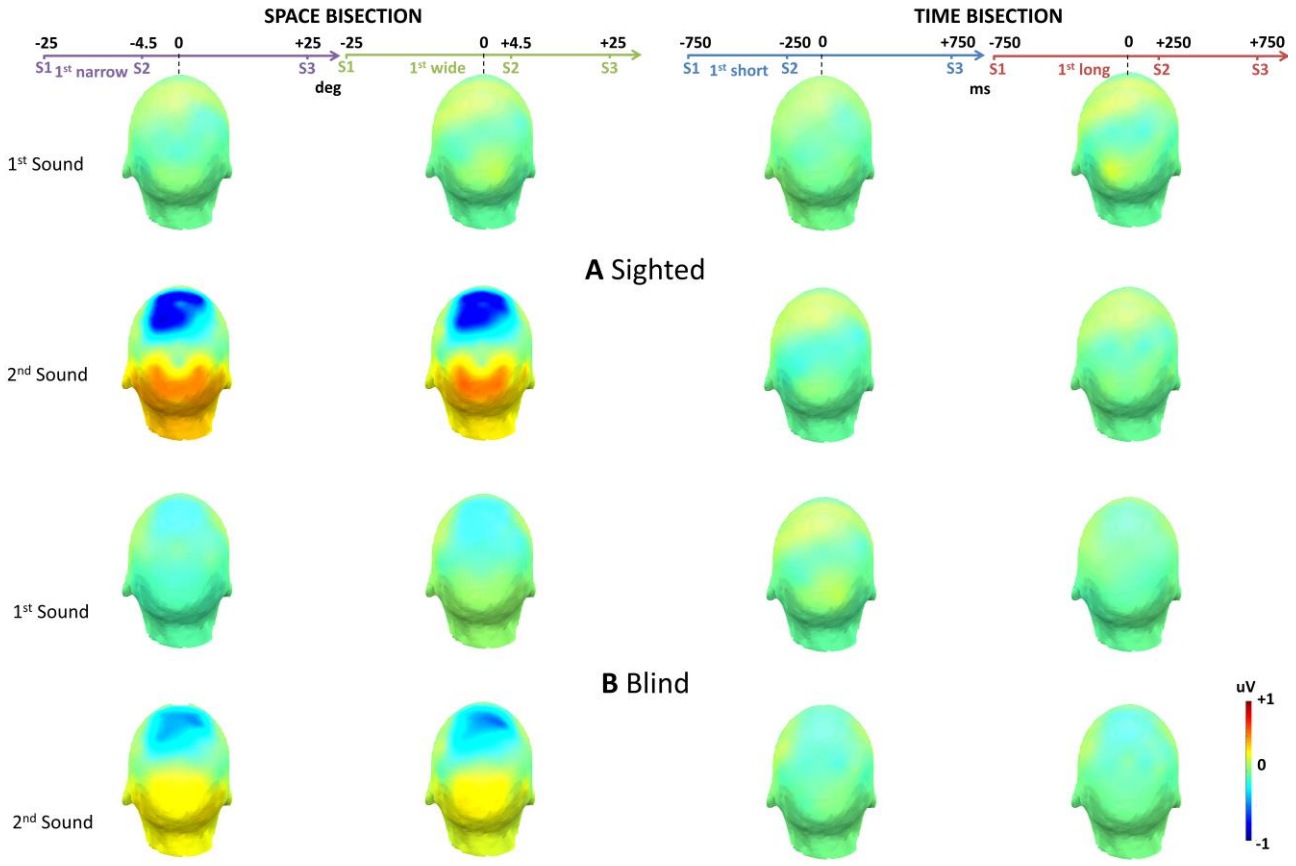

Supplementary Figure 2. ERP scalp map in the (110-160ms) time window averaged across sighted (**A**) and blind subjects (**B**). During space bisection (Left) S2 produced in sighted subjects (**A**) a positivity in parieto-occipital areas, while a negativity in front-central areas. The response was weaker in blind subjects (**B**). Both positivity and negativity were not modulated by sound position. During time bisection task (Right), a similar response was missing, as well as after S1 for both bisection tasks.

ACOP

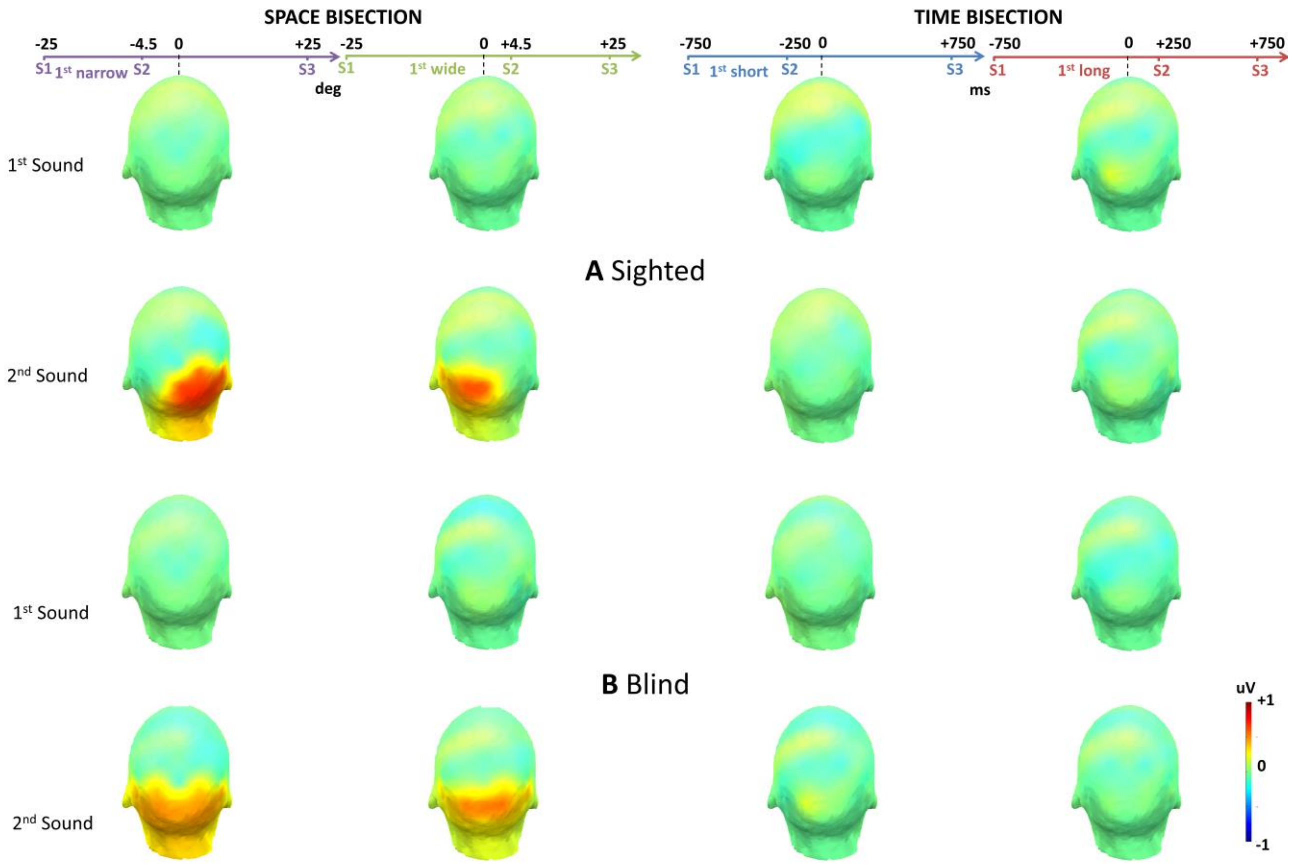

Supplementary Figure 3. ERP scalp map in the (300-500 ms) time window averaged across sighted (**A**) and blind subjects (**B**). During space bisection (Left) S2 produced a positivity in parieto-occipital areas which was contralateral to sound position in sighted subjects (**A**), while weaker and bilateral in blind subjects (**B**). A similar response was absent during temporal bisection (Right) and after S1 for both bisection tasks.

ERP before cleaning procedure

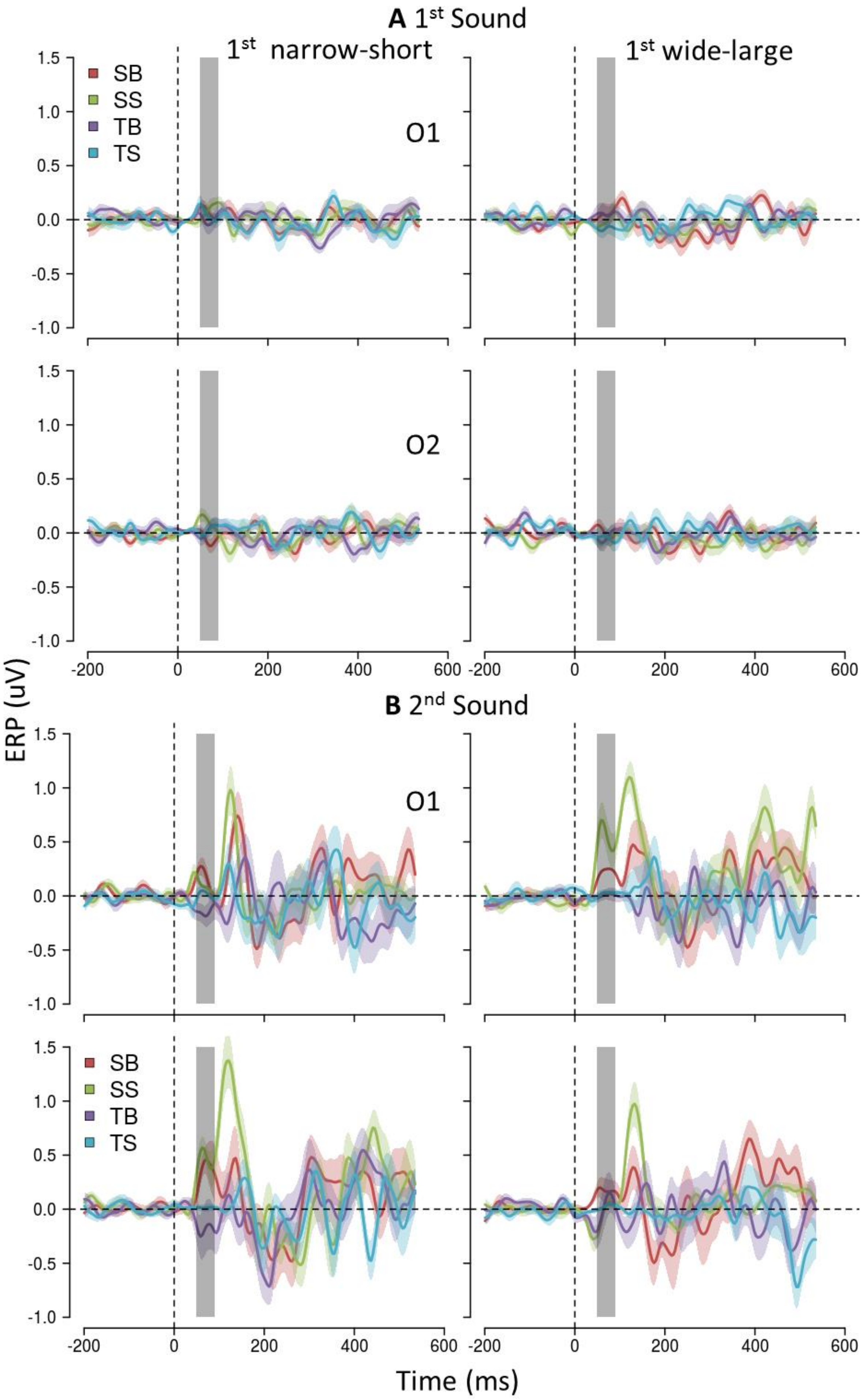

A 1st Sound
1st narrow-short
1st wide-large
SB
SS
TB
TS
O1
O2
ERP (uV)
B 2nd Sound
O1
SB
SS
TB
TS
Time (ms)

Supplementary Figure 4. Modulation of the occipital ERP response due to physical sound position considering data before cleaning procedure. **A)** Physical position of S1 does not modulate occipital response in O1 and O2. ERP (mean ± SEM) in O1, first raw, and in O2, second row, averaged across subjects. On the left, average of trials in which S2 was presented in the left hemispace (spatial bisection) or with shorter temporal separation from S1 (temporal bisection); on the right, trials in which S2 was presented in the right hemispace or with longer temporal separation from S1. Differently colored curves represent ERP responses of blind (B) and sighted subjects (S) to S1 for temporal (TB, TS) and spatial bisection task (SB, SS). $t = 0$ is first sound onset. Shaded areas delimit P70 (50-90 ms) time window. **B)** Physical position of S2 modulates occipital response of sighted individuals in O1 and O2 during space bisection task. Task related modulations are less evident with respect to cleaned data, due to the worse signal to noise ratio, but still present.

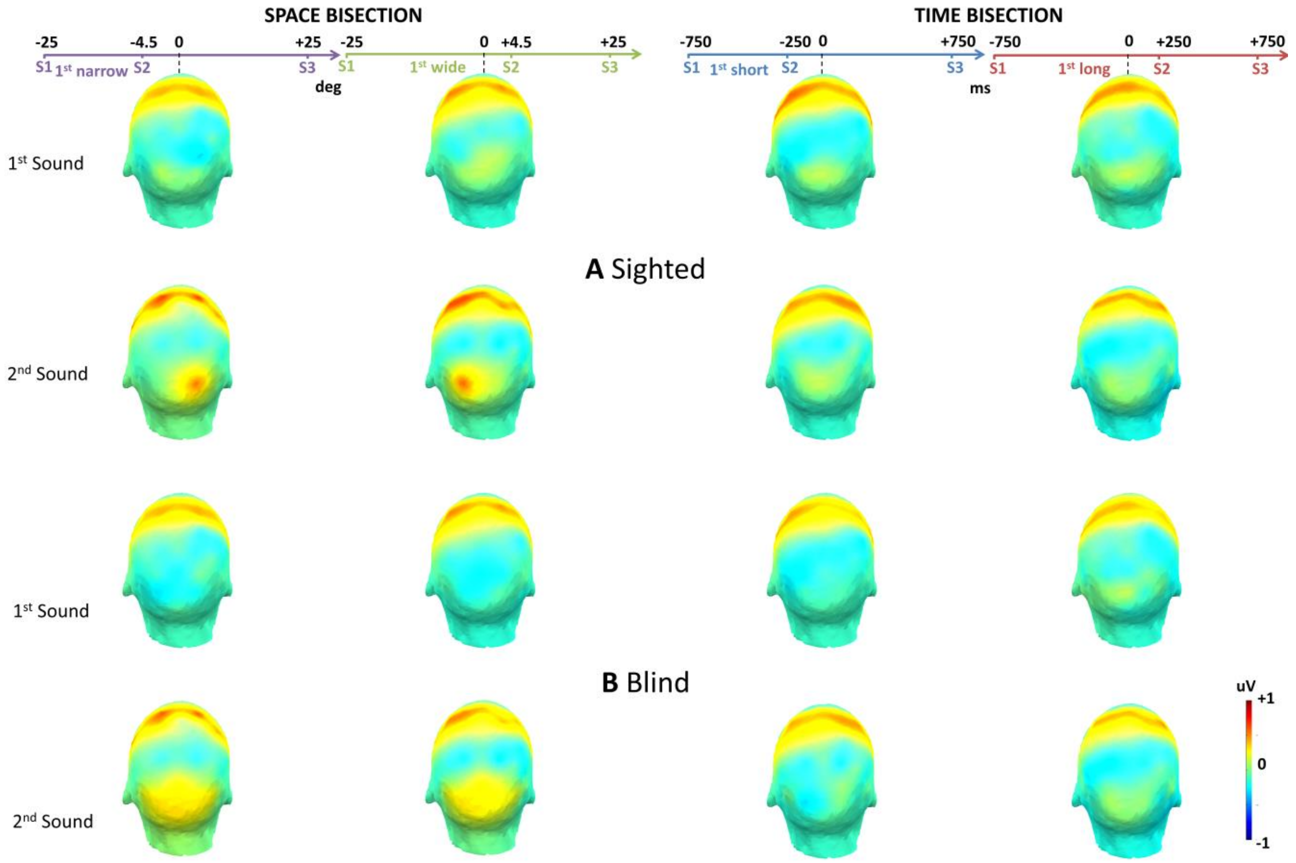

Supplementary Figure 5. ERP scalp map in the P70 time window (50-90 ms) averaged across subjects: the effect of sound and bisection task considering data before cleaning procedure. The positivity in central areas, was not modulated by any experimental factor. In sighted individuals (**A**), the parieto-occipital positivity was contralateral to S2 during the spatial bisection task (bottom left corner of **A**), when a narrow (Left) or a wide (Right) first distance corresponded to S2 from the left or in the right hemifield respectively. During time bisection task (bottom right corner of **A**), neither a short nor a long first interval in the time domain could elicit a similar response. Compared with sighted subjects, blind participants (**B**) showed during spatial bisection task a lower positivity without any contralaterality (bottom left corner of **B**). The posterior positivity was absent during time bisection task (right part of **A** and **B**) and after S1 for both bisection tasks. Importantly, no other scalp areas were involved by the early positivity. Task related modulations are less evident with respect to cleaned data, due to the worse signal to noise ratio, but still present.
